# A Course of Clinical Lectures, Delivered at the Hôtel Dieu, Paris, for the Session 1842-’43

**Published:** 1843-06-10

**Authors:** A. F. Chomel

**Affiliations:** Paris


### THE MEDICAL EXAMINER,

### And Retrospect of the Medical Sciences.

Vol. VI.] PHILADELPHIA, SATURDAY, JUNE 10, 1843. [No. 11.

### A COURSE OF CLINICAL LECTURES,

*Delivered at the Hôtel Dieu, Paris, for the Session* 1842–'43.

BY A. F. CHOMEL, M. D.

### LECTURE IX.

In the resumé which I am about to present to you of the more interesting cases which we have had to treat in the course of the present year,—that is to say, from March, 1842, to March, 1843,—I shall accompany the rapid history of the several facts with some practical remarks which may be instructive to you. I shall commence with the cases of typhoid fever.

Since the 15th of March, 1842, to the month of March of the present year, we have had one hundred cases of typhoid fever. Out of this number twenty have died, or one-fifth. The mortality was much greater in winter than in summer. Out of seventy-two cases which occurred during the summer, only fourteen died. This is a result directly opposed to that which the statistics of pneumonia presents, where the mortality is proportionally much greater in summer than in winter, as we shall see hereafter.

There are in our wards twenty beds for men, and thirty-two for women, including twelve beds for convalescents. In spite of this disproportion, the cases of typhoid fever were much more numerous among the men than the women; for among the former there were fifty-nine, and forty-one among the second. As a general rule, women are much less subject to contract this affection than men. Out of these fifty-nine male patients fifteen succumbed; whilst of the forty-one women, only five died.

In recapitulating the cases of typhoid fever that we have had to treat in the course of the last eleven years, we can count five hundred and twelve. This *relevé* has enabled us to ascertain the mean age at which persons are attacked with typhoid fever to be from fifteen to twenty years. The mortality is one-sixth from twenty to thirty-five years. Typhoid fever rarely happens over the latter age. Out of the hundred cases which we have had during the past year, the disease was developed in thirty cases during the first year's residence in Paris. Of these thirty, three only died. It is observed, generally, however, that this fever is more frequently fatal among such, than those who are attacked at a later period. With regard to *duration*, the mean time has been, in general, about thirty days for the severe cases, and five days only for lighter cases. When the *invasion* is sudden the mortality is less, whilst, when the disease developes itself slowly after a long incubation, it is usually much more mortal. The appearance of the typhoid eruption is very frequent; indeed, it is, as you know, one of the characteristic signs of the disease. The *petechiæ*, very different from the hemorrhagic spots, with which they should not be confounded, are not a symptom of any gravity, whilst the latter, on the contrary, indicate great danger. Two cases presented this symptom in the course of the year, and both of these died. Delirium, when violent, is also a symptom of much gravity. This symptom occurred in nine cases out of one hundred. A comatose state also is of equally bad augury. The same may be said of intestinal hemorrhage. In the preceding year hemorrhage from the bowels was the cause of death in one-half the patients that we lost. The same proportion, in this respect, has occurred the present year. The sloughs which occur sometimes over the sacrum, indicate also great danger; first, because they announce a general feebleness, or prostration of the forces, and finally, because they are ordinarily connected with paralysis of the bladder and rectum. The mortality in such cases is also about one-half. Perforation of the intestine has happened three times this year, and the patients died from a partial consecutive peritonitis. Perforation occurred in one case, a woman, on the eighteenth day of the disease; in another patient, it occurred just as he entered on convalescence; in the third, it happened on the twenty-first day. Although exceedingly grave, and generally mortal, it is not invariably fatal. When the perforation is small, and but a small quantity of matter escapes by the opening, the partial peritonitis which results may be cured.

*Complications.*—In one patient abscesses occurred, which terminated in death; another was attacked with erysipelas; and a third with pericarditis, equally fatal. Some cases were complicated with intense acute bronchitis. One woman had *phlegmasia alba dolens.* Eleven patients had the face injected; the pulse full and strong, with bleeding at the nose. These were all treated with antiphlogistics, and one only died. These cases were evidently those of the inflammatory fever of Pinel. In four cases the nervous phenomena predominated over the others; in these there was subsultus, &c. This ataxic form is, as you know, one of the most fatal of the disease; three out of the four died. Three others presented an adynamic form, and some a mixed type—ataxo-adynamic. All these cases were of the gravest nature. Of the remaining sixty-six cases, of which thirty-six were light, there was nothing of particular interest.

*Treatment.*—With regard to the treatment of typhoid fever, which I can only touch upon, I cannot abstain from remarking what I have said so frequently before—that it is very difficult to appreciate in a just and true manner by the treatment, the action of the different remedies that we have employed, and that we still employ in the treatment of this affection. I have myself experimented largely with different remedies, more particularly with carbonic acid and the chlorides. The first results were in both cases equally happy and satisfactory; beyond, indeed, our hopes, so that we commenced hoping that we had at last found efficacious means; but before long we were disabused; for reverses soon followed to balance our successes, and we finished by being convinced that these agents could have a certain favourable influence according to circumstances, but that they were by no means heroic. I believe that the same results followed trials made in other hands. You must not lose sight of the fact that we have reference to a disease of the gravest nature, which, whatever some physicians may say, has existed from time immemorial, and upon which any number of therapeutic essays have been made, by each school, and by each man who entertained any preconceived notions of the nature or elements of the disease. The ancients, regarding the fœtid nature of the discharges which occur in typhoid fever, used means which were calculated, according to them, to modify the putridity of the disease—and they accordingly purged freely. More recently, the physicians of the last century, believing that there was some peculiar bilious element, gave emeto-cathartics; thus Tissot employed this medication in the epidemic at Lausanne, which he called bilious fever, but which was evidently nothing else but the typhoid affection, with predominance of the bilious element. With this treatment that distinguished physician obtained great success; however, as there reigned at this time an epidemic in Switzerland, and you know that the success which certain medicines obtain in epidemic affections do not realize expectations always in the same diseases, when sporadic; so that the treatment of Tissot was soon abandoned. At a period still more remote, when the chemical doctrines were high in favour, all the vital actions of the economy were considered as mere chemical phenomena; and an attempt was made to apply these notions in the practice of medicine. With this hypothesis, they believed that putridity was the chief element in typhoid fever, and substances were sought for to neutralize this putridity. In consequence of this idea, recourse was had to camphor, and other remedies supposed to be antiseptic. Success attended sometimes this practice, but failure was also very common; the insufficiency of the remedy was acknowledged, and it was abandoned along with the others, and new ones were sought for. To these humoral and chemical schools succeeded that of Pinel, who gave to the disease that we now call typhoid fever, the name of adynamic fever; and in conformity to the idea of this designation, he proposed the use of tonics. It should be mentioned that Pinel practised at this time at Bicètre, and saw this disease accompanied by so much prostration and feebleness, that he was in some measure authorised in considering it as an asthenic affection, and giving to it the name of adynamic fever. Besides, the description which he has left leaves no doubt upon the nature of the disease he so denominated—typhoid fever of a very low type, such as we frequently meet with in old persons. This modification in the pathological ideas, necessarily caused some modification in the therapeutic notions, and all those physicians who followed his principles adopted his practice, and gave tonics. It should be stated that the tonics employed by Pinel were not of the most energetic character; they were mild and unirritating in their nature, so that, in reality, he did not treat typhoid fever very differently from the other physicians of his period. At this time great hygienic improvements were introduced into the hospitals. Patients who before slept two in a bed, now had a single bed appropriated to each, and much greater cleanliness was observed. This caused general amelioration in all the low forms of disease.

At the very moment when the school of Pinel was in the zenith of its glory, Broussais appeared, who, adopting an entirely different doctrine, attacked the medical and therapeutical system of Pinel, and substituted for the name of adynamic fever, that of gastro-enteritis, looking upon that as a mere inflammation of the gastro-intestinal tube, which his predecessors had considered a complex morbid state of the system.

He inveighed with great force against the use of tonics, and admitted in the treatment of this affection the antiphlogistic remedies alone. This doctrine prevailed, in its turn, for some time, and the system of bloodletting was pushed so far that we have seen some of its unfortunate victims expiring with their bodies covered with leeches. Bloodletting had become a general rule in almost all diseases. But this system, at variance with the most generally received principles of sound therapeutics, could not last long; it fell through at the appearance of a more sound and prudent system: and the former system ought, by all means, to be banished in the treatment of a disease such as typhus fever, which assumes divers forms, presenting themselves with very different modifications, the treatment of which must consequently vary very much.

There are remedies which we can employ without any danger in typhoid fever, such as carbonic acid and the chlorides, at the same time that we have recourse to a rational treatment; for there exists in these diseases a deleterious principle, against which we must strive to contend by specific remedies.

The rational treatment consists in hygienic remedies and medicinal remedies, properly so called. The first may be very advantageous when properly directed; thus cleanliness alone may be extremely beneficial. It suffices to have seen the patients in the hospitals, before the reform which has since been made in these establishments, to be convinced of the happy influence of good hygienic measures.

The influence of the hygienic remedies is immense in all diseases, and principally in that of which we are now treating. To the ordinary hygienic precautions, we must also add the moral ones, the removal of everything tending to disturb or disquiet the patient, the consolations which friendship or even mere humanity may procure, etc. These have a very great influence, also, on the happy issue of the disease. The advantages derived from them appear to us so great, that it would, perhaps, be better for the patient to be deprived of the therapeutical remedies, than to want the proper hygienic care and suitable moral resources. We therefore place them first; they are, moreover, remedies which are always suitable, and which may be applied in all the different forms of the disease.

The medicinal remedies constitute, or ought to constitute, the rational system of medicine which we must follow in all diseases, modifying it according to circumstances, and according to the different forms which diseases assume. To wish to adopt one uniform system in almost all diseases, admitting even that they have a phlogistic state of the system as a base, as, for example, measles, scarlatina, &c., would be a grievous error; for many divers systems may present themselves in the course of these diseases, which, however inflammatory they may really be, are symptoms, nevertheless, which will oblige us to modify our treatment, without even taking into consideration the variations respecting individuals, age, climate, or a number of other circumstances. How can we imagine that there should be but one method for treating all these? This is, nevertheless, the opinion of some physicians, who absorbed by the one idea of inflammation, rely entirely on the efficacy of bloodletting and antiphlogistic remedies.

The skilled practitioner, also, will never adopt a general and absolute formula, for he will find himself under the necessity of changing his formulæ every instant. To impress more forcibly the value of this precept, it will suffice to glance at the variety and multiplicity of forms assumed by typhoid fever.

Typhoid fever presents itself often with all the symptoms indicating inflammation; the pulse is full and frequent; great heat of skin; the face is of a dusky hue; stupor; the eyes glazed, etc. We have present what the ancients termed inflammatory fever, synocha. In this case, the antiphlogistic remedies are indicated without doubt, and they will have to be repeated according to circumstances. This is the inflammatory form which typhoid fever sometimes assumes, and in which, I repeat it, there is every indication for the antiphlogistic remedies, especially in the first stage, when the strength of the patient has not yet been prostrated; for this is a circumstance demanding a great deal of attention. We are not sufficiently authorised to employ this treatment because the disease has presented some symptoms of inflammation, if, on the other hand, the morbid state of the system has lasted several days, and if the patient evinces already a considerable prostration; we must then abstain from bloodletting, and confine ourselves to remedies indirectly antiphlogistic and less active.

In other cases, this affection is accompanied, from the very onset, by bilious symptoms; the mouth has a bad appearance; tongue covered with a yellowish coat; bilious vomitings; the face has an icteric tint. This is again one of the particular forms of typhoid fever, that which Tissot treated very successfully by purgatives. This is a case where we may have recourse to emetics, emeto-cathartics, and to acidulated drinks. At other times it assumes the adynamic form. The adynamic symptoms are of two kinds, each requiring a different treatment. Thus we can have weakness, with all the signs of the greatest vital depression; the skin is below the normal temperature; the pulse is feeble and very slow; stools involuntary; the patient is in a state of stupor, almost unconscious of what is transacting around him. In such a case we must employ the tonics in large doses; we must raise his strength at all hazards, and save his life. To accomplish this, we may give him the wine of bark or Madeira wine, in divided doses, frequently repeated; I have thus prescribed as much as half of a bottle a day with the greatest success. I have, by these means, snatched from the grave a young girl, who was cold, in a state of the most extreme prostration, and who, in a word, appeared to be breathing her last. We gave her tonics in divided doses, gradually increasing. In a few days she regained her strength, her pulse grew stronger, and gradually she recovered. Tonics, then, are very beneficial in such cases. If, on the contrary, notwithstanding the apparent state of prostration, the pulse retains a certain force, and if the heat be great, tonics will not have the great efficacy they possessed in the preceding case; they may raise, for a few minutes, the patient's sinking strength, they may afford a momentary increase of vitality, but the disease will still go on, and its progress will sometimes be the more rapid; so that it is more prudent in doubtful cases to abstain from powerful tonics, for fear of aggravating the disease.

The ataxic form which typhoid fever assumes, is one of the most serious and most fatal. When there is much nervous disturbance, and the intellectual faculties greatly involved—when the memory fails, stupor supervenes, coma, and subsultus tendinum, there is great danger. The patients thus affected often sink on the ninth or tenth day, and the resources of art prove generally completely ineffectual in this condition of things.

If, in the case just mentioned, a sufficiently active febrile reaction supervenes, there would be indications for bloodletting; often with the assistance of this last remedy all the nervous phenomena are very much improved.

In some cases the ataxic form is accompanied by the adynamic; we have, then, what are termed the ataxo-adynamic symptoms, and the tonics, prudently administered, may be very beneficial; but, unfortunately, these rarely succeed in such cases.

We have, finally, mixed nervous forms, where we are obliged to employ an entirely empirical treatment, and to use the different antispasmodic remedies. The use of the baths sometimes causes a very great improvement of the nervous symptoms; musk and castor may also prove beneficial, but their action is uncertain: and we never feel confident that the changes following the administration of these remedies result from the action of the remedies themselves, there is so great an irregularity in the progress of the disease. The same remedy which appears to have been useful in one case, does not act, or acts injuriously in another, if we judge of it by its effects. The application of remedies in this form of the disease is very difficult.

This ataxo-adynamic form is really a desperate one for physicians, and often a fatal one for the patients.

The baths, as I have already mentioned, and the application of cold water to the head may afford some relief; but when the danger is very great we are authorised to try everything, even those remedies whose action is most uncertain, conformably to the axiom: Melius anceps quàm nullum. It is, for instance, in a case of this kind that we may employ the musk. I have seen some very happy effects produced by this medicine, especially on a child who was in a profound coma, with stiffness of the limbs, resembling that of tetanus, whose life was despaired of, and who was cured under the use of this medicine. But its action is always empirical, and we must always consider it as such, and never pretend to anticipate its effects, at least with any degree of certainty.

*Paris, March,* 1843.

